# Chemical Survey of Three Species of the Genus *Rauhia* Traub (Amaryllidaceae)

**DOI:** 10.3390/plants11243549

**Published:** 2022-12-16

**Authors:** Luciana R. Tallini, Edison H. Osorio, Strahil Berkov, Laura Torras-Claveria, María L. Rodríguez-Escobar, Francesc Viladomat, Alan W. Meerow, Jaume Bastida

**Affiliations:** 1 Departament de Biologia, Sanitat i Medi Ambient, Facultat de Farmàcia i Ciències de l’Alimentació, Universitat de Barcelona, Av. Joan XXIII 27–31, 08028 Barcelona, Spain; 2 Facultad de Ciencias Naturales y Matemáticas, Universidad de Ibagué, Carrera 22 Calle 67, Ibagué 730001, Colombia; 3 Institute of Biodiversity and Ecosystem Research at the Bulgarian Academy of Sciences, Department of Plant and Fungal Diversity, 23 Acad, G. Bonchev Str., 1113 Sofia, Bulgaria; 4 School of Life Sciences, Arizona State University, Tempe, AZ 85282, USA

**Keywords:** acetylcholinesterase, Alzheimer’s disease, Amaryllidaceae, alkaloids, galanthamine, *Rauhia*

## Abstract

Plant biodiversity is an important source of compounds with medicinal properties. The alkaloid galanthamine, first isolated from *Galanthus woronowii* (Amaryllidaceae), is approved by the FDA for the palliative treatment of mild to moderate Alzheimer’s disease due to its acetylcholinesterase (AChE) inhibitory activity. Obtaining this active pharmaceutical ingredient, still sourced on an industrial scale from the Amaryllidaceae species, is a challenge for pharmaceutical companies due to its low natural yield and the high cost of its synthesis. The aim of this work was to determine the alkaloid profile of three different *Rauhia* (Amaryllidaceae) species collected in Peru, and to assess the potential application of their extracts for the treatment of Alzheimer’s disease. The alkaloids were identified by gas chromatography coupled to mass spectrometry (GC-MS), and the AChE inhibitory activity of the extracts was analyzed. Thirty compounds were quantified from the *Rauhia* species, the *R. multiflora* extract being the most interesting due to its high diversity of galanthamine-type structures. The *R. multiflora* extract was also the most active against AChE, with the half maximal inhibitory concentration (IC_50_) values of 0.17 ± 0.02 μg·mL^−1^ in comparison with the IC_50_ values of 0.53 ± 0.12 μg·mL^−1^ for galanthamine, used as a reference. Computational experiments were carried out on the activity of the galanthamine-type alkaloids identified in *R. multiflora* toward five different human AChE structures. The simulation of the molecules 3-*O*-acetylgalanthamine, 3-*O*-acetylsanguinine, narwedine, and lycoraminone on the 4EY6 crystal structure theoretically showed a higher inhibition of hAChE and different interactions with the active site compared to galanthamine. In conclusion, the results of this first alkaloid profiling of the *Rauhia* species indicate that *R. multiflora* is an important natural source of galanthamine-type structures and could be used as a model for the development of biotechnological tools necessary to advance the sustainable production of galanthamine.

## 1. Introduction

According to the World Health Organization (WHO), important medical and pharmacological discoveries are made through a greater understanding of the Earth’s biodiversity [1]. Nature is a source of natural products and/or natural product structures that play a significant role in the search for new drugs [2]. Alkaloids, nitrogenated compounds metabolized mainly by plants, are of particular interest in the development of new medicines due to their structural diversity [3,4]. The plant family Amaryllidaceae, specifically the Amaryllidoideae subfamily, contains exclusive isoquinoline alkaloids known as Amaryllidaceae alkaloids, which show remarkable biological activities [5]. This subfamily contains more than 800 species, which are classified into 59 genera and distributed in different climatic zones, including South America [6].

The subfamily Amaryllidoideae has been the focus of diverse publications in recent decades, which have provided new information about its botanical, chemical, and biological characteristics. Galanthamine—an alkaloid first isolated from *Galanthus woronowii* (Amaryllidaceae)—together with donepezil and rivastigmine are the only three acetylcholinesterase inhibitor drugs approved by the Food and Drug Administration (FDA) for the palliative treatment of Alzheimer’s disease [7]. These products are able to inhibit the acetylcholinesterase enzyme, thus increasing the presence of acetylcholine, a neurotransmitter involved in the process of learning and memory, in the human brain [7]. The screening of different Amaryllidaceae species and the search for Amaryllidaceae alkaloids with acetylcholinesterase inhibitory activity has increased in the last few years.

*Rauhia* (Amaryllidaceae) is a small, xeromorphic genus established by Traub (1957) [8] with the description of *R. peruviana* Traub (1957). Ravenna (1969) [9] recognized that *Rauhia peruviana* was conspecific with *Phaedranassa multiflora* Kunth (1850) and established the combination *Rauhia multiflora* (Kunth) Ravenna. *Rauhia megistophylla* (Kraenzlin) Traub (1966) [10] is a synonym of *R. multiflora*. Four additional species have since been described: *Rauhia staminosa* Ravenna (1978), *Rauhia decora* Ravenna (1981), *Rauhia occidentalis* Ravenna (2002), and *Rauhia albescens* Meerow & Sagást. (2019) [11,12,13,14]. All the species have greenish-white to green flowers and produce deciduous, carnose, pseudo-petiolate leaves either contemporaneously with the flowers or emerging from the large, globose bulbs with the scape. Young leaves are sometimes tessellated. The fruit is a tri-loculicidal capsule releasing numerous flat, papery, winged blackish-brown seeds that are probably wind dispersed.

The genus *Rauhia* is endemic to the seasonally dry Marañón woodlands of the inter-Andean valleys of northern Peru and is the first branch of the tribe Eucharideae in phylogenomic analyses [15]. The Eucharideae is a well-supported assemblage of six genera belonging to the Andean tetraploid clade of the American Amaryllidaceae [15,16], distinguished by their pseudo-petiolate leaves, the loss or pseudogenization of much of the ndh family of plastid genes, and 2n = 46 chromosomes. *Rauhia* is closely related to the genera *Eucrosia* Ker Gawler, *Phaedranassa* Herb., and the monotypic *Plagiolirion horsmanii* Baker [15]. *Rauhia* species typically form small to moderate populations growing among Cactaceae and/or seasonally deciduous trees and shrubs. The bulbs are dormant during the long dry season, though the desiccated leaves sometimes remain visible above ground. Nothing is known about their pollination biology.

**Figure 1:**
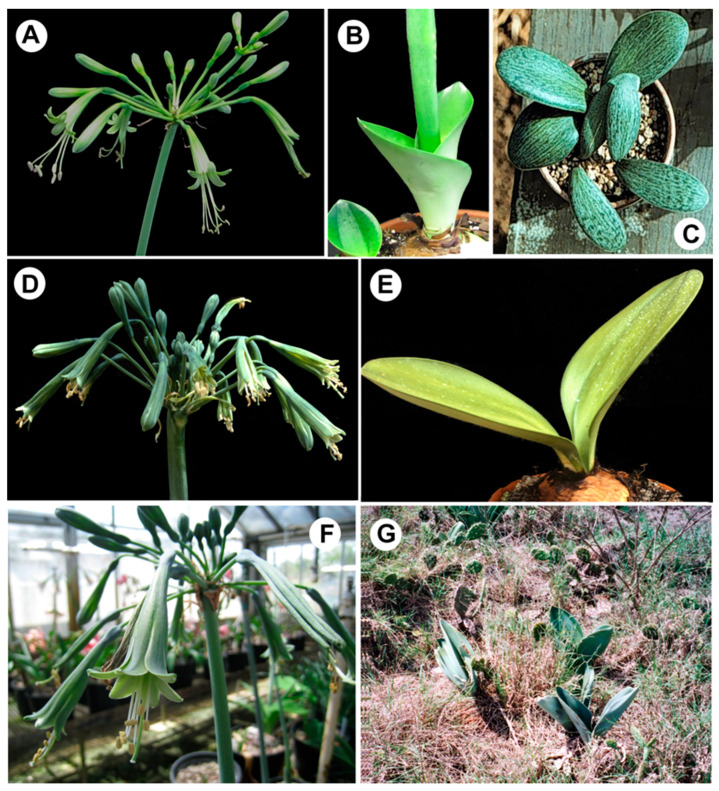
*Rauhia* species investigated in this study. (**A**–**C**). *R. decora*. (**A**). Flowers. (**B**). Emerging leaves with induplicate vernation that form a funnel-like structure, putatively a device for channeling moisture to the bulb. (**C**). Juvenile leaves showing tessellation. (**D**,**E**). *R. multiflora*. (**D**). Flowers. (**E**). Fully developed leaves. (**F**,**G**). *R. staminosa*. (**F**). Flowers. (**G**). In natural habitat, Amazonas region, Peru. Photo credits: (**A**,**B**,**D**–**F**), Alan Meerow. (**C**) Dylan Hannon. (**G**) Henk van der Werff.

*R. multiflora* is found in the region of Cajamarca in the province of Jaen, Peru, at a 500–800 m altitude, whereas *R. decora* was described from the region of Amazonas, on the west side of the Utcubamba River valley between Bagua and Chachapoyas, with no altitude reported. The live material examined in this study was collected at 500 m. *R. staminosa* is found not far from *R. decora*, also in Amazonas, between Bagua and Tingo on the way to Chachapoyas, with no altitude reported in the protologue. The live material examined here was collected at 800 m. *R. occidentalis* was described from Cajamarca, the province of Chilete, Choropampa, near mount Palco. Ravenna (2002) considered it to be intermediate between *R. multiflora* and *R. staminosa*. *R. albescens* was found in the La Libertad region, Pataz Province, near Huaylillas, at 2300 m, which means this is the most southern species studied and grows at the highest altitude (Figure 1).

The aim of this work was to evaluate the diversity of alkaloids in the bulb extracts of three species of the genus *Rauhia* collected in Peru and to verify their potential use in Alzheimer’s disease therapy. The species *R. staminosa*, *R. decora*, and *R. multiflora* were analyzed by gas chromatography coupled to mass spectrometry (GC-MS) and in vitro and in silico experiments were carried out to analyze their acetylcholinesterase inhibitory activity.

## 2. Results and Discussion

### 2.1. Alkaloid Profiling

**Figure 2:**
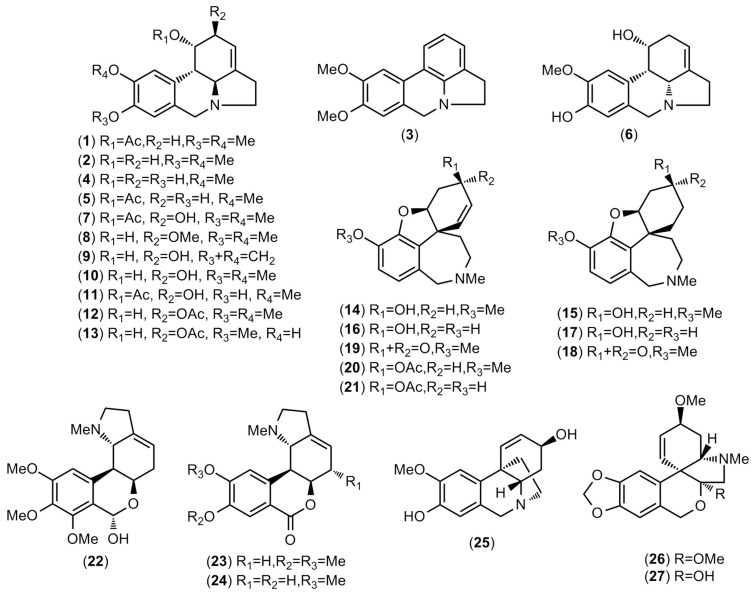
Alkaloids identified in *Rauhia* species by GC-MS.

GC-MS analysis revealed 30 alkaloids in the species *R. staminosa*, *R. decora*, and *R. multiflora*, three of which were not identified (Table 1 and Figures S1–S3). Each alkaloid described in Table 1 was quantified as a μg of galanthamine (GAL), which was related to the mg of the dry weight (μg GAL·100 mg^−1^ DW). The identified structures are presented in Figure 2.

More than 650 Amaryllidaceae alkaloids are reported in the literature [17] and their structures are classified into 42 skeleton types, among which lycorine, haemanthamine, homolycorine, galanthamine, and pretazettine are among the most representative [17]. As shown in Table 1, the alkaloids identified in the *Rauhia* species in the present study are cataloged according to the scaffold type.

In *R. staminosa* (sample A), high amounts of Amaryllidaceae alkaloids (409.3 μg GAL·100 mg^−1^ DW) were quantified and 13 lycorine-type structures (317.5 μg GAL·100 mg^−1^ DW) were identified. Compounds **12** and **13**, identified as 2-*O*-acetyl-9-*O*-methylpseudolycorine (168.9 GAL·100 mg^−1^ DW) and 2-*O*-acetylpseudolycorine (38.2 μg GAL·100 mg^−1^ DW), were predominant. Galanthamine- and homolycorine-type scaffolds were also detected in this species. Two unidentified structures, **29** and **30**, were observed and, based on the fragmentation pattern, the former can be classified as a homolycorine-type alkaloid (Table 1).

Among the species listed in Table 1, *R. decora* (sample B) had the highest diversity of the Amaryllidaceae alkaloid groups, with lycorine-, galanthamine-, homolycorine-, haemanthamine-, and pretazettine-type skeletons being detected. The most prevalent was the lycorine-type alkaloid, identified as 2-*O*-acetyl-9-*O*-methylpseudolycorine (**12**) (135.6 μg GAL·100 mg^−1^ DW). One unidentified structure was found in this species, which exhibited the usual fragmentation pattern of homolycorine-type alkaloids.

Promising results were obtained for the species *R. multiflora* (sample C), which showed a high diversity and amounts of galanthamine-type alkaloids (278.7 μg GAL·100 mg^−1^ DW) (Table 1 and Figure 2). Galanthamine (**6**) was the predominant alkaloid quantified in *R. multiflora* (103.6 μg GAL·100 mg^−1^ DW), followed by lycoramine (**7**) (73.1 μg GAL·100 mg^−1^ DW). Additionally, one pretazettine-type structure was quantified (9.9 g GAL·100 mg^−1^ DW).

The alkaloid galanthamine has been used for the palliative treatment of mild to moderate symptoms of Alzheimer’s disease since 2001 [18,19]. Different Amaryllidaceae plants metabolize galanthamine, the species *Narcissus* cv Carlton, *Leucojum aestivum*, and *Lycoris radiada* being the principal sources for pharmaceutical companies [20]. Additionally, high concentrations of galanthamine have been described in an in vitro culture of *Hippeastrum papilio* (Amaryllidaceae), patented under the number EP2999480B1 [21].

The presence of substantial amounts of other types of Amaryllidaceae alkaloids, especially lycorine, can be a hindrance for the industrial process of the purification of galanthamine [20,22]. In the preparation of galanthamine as an active pharmaceutical ingredient, no lycorine should remain due to its cytotoxicity [23,24]. The low natural yield of galanthamine and the costly and time-consuming processes required for its industrial-scale production call for the development of efficient tools that can control its biosynthesis [25].

Among the results presented in Table 1, the species *R. multiflora* stands out for its high diversity of galanthamine-type alkaloids and different unquantified Amaryllidaceae alkaloid scaffolds, especially lycorine-type structures. This species may therefore be a suitable candidate for use as a model plant to elucidate the biosynthesis of galanthamine-type alkaloids. The knowledge generated would contribute to developing new biotechnological approaches for the sustainable and scaled-up production of galanthamine.

### 2.2. Acetylcholinesterase Inhibition

**Figure 3:**
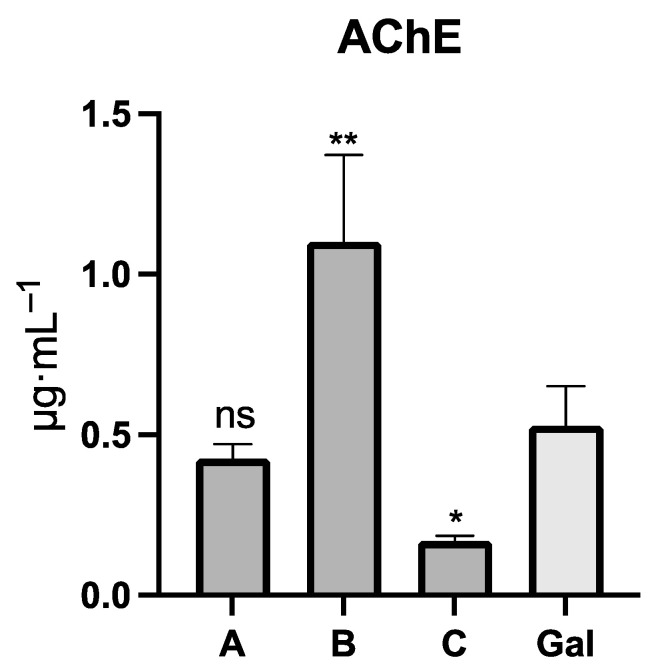
AChE inhibitory potential of *Rauhia* species. A = *R. staminosa*; B = *R. decora*; C = *R. multiflora*; Gal = galanthamine; ** *p* < 0.01, * *p* < 0.1, and *ns* (not significant).

The acetylcholinesterase (AChE) inhibitory activity of the *Rauhia* species was evaluated (Figure 3). *R. multiflora* showed the half maximal inhibitory concentration (IC_50_) values of 0.17 ± 0.02 μg·mL^−1^, while *R. staminosa* and *R. decora* presented IC_50_ values of 0.43 ± 0.05 and 1.10 ± 0.27 μg·mL^−1^. Galanthamine, used as a positive control, exhibited IC_50_ values of 0.53 ± 0.12 μg·mL^−1^ (Table 1 and Figure 3).

The high amount and diversity of galanthamine-type alkaloids detected in *R. multiflora* are likely responsible for the high AChE inhibitory activity of the extract (Table 1). Some authors have evaluated the inhibitory activity of sanguinine (**16**) and galanthamine (**14**) against *Electrophorus electricus* AChE (*Ee*AChE), which showed IC_50_ values of 0.10 ± 0.03 and 1 ± 0.05 μM, respectively [26]. The potential of the alkaloids narwedine (**19**), lycoramine (**15**), and lycoraminone (**18**) against human erythrocyte AChE (*h*AChE) have also been reported in the literature, showing IC_50_ values of 282 ± 33, 456 ± 57, and >500 μM, respectively [27,28].

Many studies have described the alkaloid profiles and AChE inhibitory properties of different Amaryllidaceae species from South America, but none of the published results match the findings reported here for *R. multiflora*. Despite differences in the extract preparation, it is of interest to briefly review the most important results reported in the literature on this topic.

**Figure 4:**
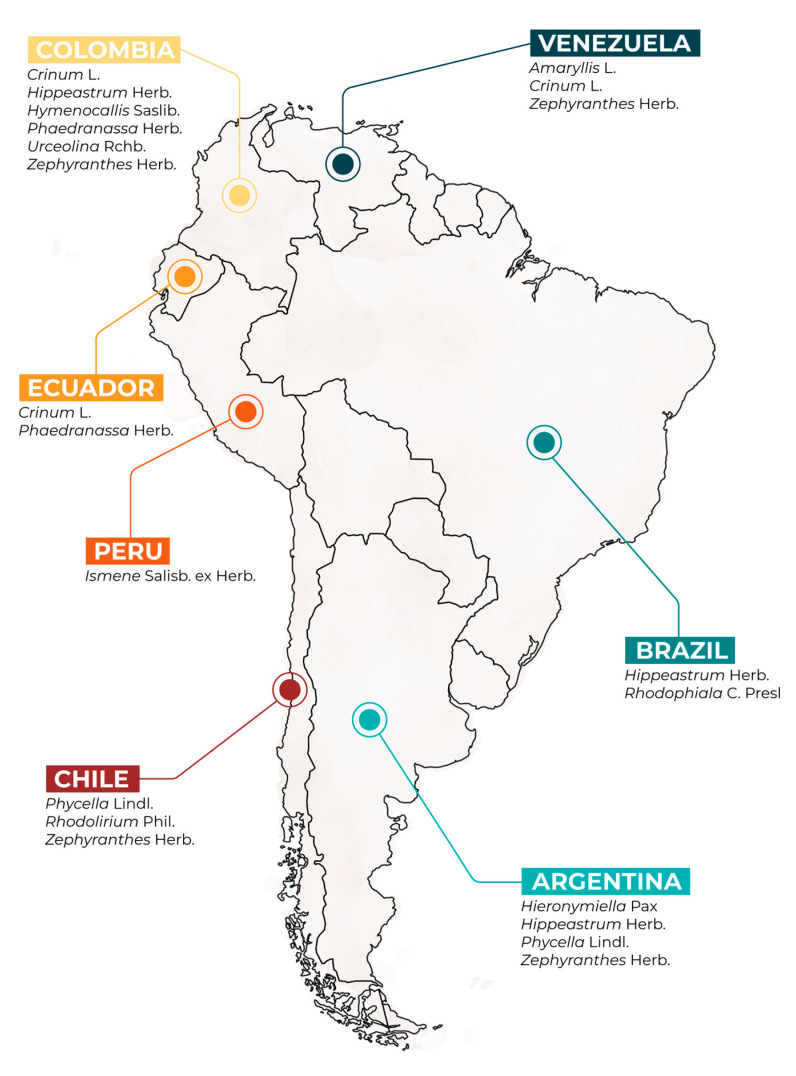
Map of South America showing Amaryllidaceae current genera whose alkaloid profiles and acetylcholinesterase inhibitory activity have been studied recently in this region.

Among species from the genera *Crinum*, *Eucharis*, *Hippeastrum*, *Hymenocallis*, *Phaedranassa*, and *Zephyranthes* collected in Colombia, Eucharis bonplandii (Kunth) Traub was the most active against *h*AChE, with IC_50_ values of 0.72 ± 0.05 μg·mL^−1^, and was found to contain lycorine-, haemanthamine-, and galanthamine-type structures, the latter represented by galanthamine-*N*-oxide, sanguinine, galanthamine, and narwedine [29,30,31,32]. In recent studies on six species of *Phaedranassa* and the species *Crinum* x *amabile* Donn collected in Ecuador, *Phaedranassa cuencana* Minga, C. Ulloa, and Oleas was the most active against *Ee*AChE, with IC_50_ values of 0.88 ± 0.11 μg·mL^−1^; three galanthamine-type alkaloids were detected: galanthamine, sanguinine, and *N*-demethylgalanthamine [33,34,35]. The plant *Ismene amancaes* (Ker Gawl.) Herb. collected in Peru showed a low activity against *Ee*AChE, with IC_50_ values of 14.6 ± 0.6 μg·mL^−1^, although high concentrations of lycoramine, a galanthamine-type alkaloid, were detected [36]. Among different species of the genera *Rhodophiala*, *Rhodolirium*, and *Phycella* collected in Chile, the bulb extract of *Rhodophiala splendens* (Renj.) Traub was the most active against *Ee*AChE, with IC_50_ values of 3.62 ± 0.02 μg·mL^−1^, although no galanthamine-type alkaloid was reported in this plant [32,37,38,39]. A study on the genera *Amaryllis*, *Zephyranthes*, and *Crinum* collected in Venezuela found the strongest *Ee*AChE inhibitory activity in a *C. amabile* extract, with IC_50_ values of 0.88 μg·mL^−1^ [40]; the main alkaloids found in the extract were of the crinine/haemanthamine type, and sanguinine, a galanthamine-type alkaloid, was also detected [40]. Among nine species of the genera *Hippeastrum* and *Rhodophiala bifida* (Herb.) Traub, all collected in Brazil [41,42], the species *Hippeastrum papilio* (Ravenna) Van Scheepen and *Hippeastrum glaucescens* (Mart. ex Schult. & Schult. f.) Herb. were the most active against *Ee*AChE, with IC_50_ values from 0.33 to 0.49 μg·mL^−1^, and galanthamine was the main constituent in both extracts [41]. Species from the genus *Habranthus*, *Hieronymiella*, *Hippeastrum*, *Phycella*, and *Rhodophiala*, all collected in Argentina, were investigated as possible sources of cholinesterase inhibitors [26,43,44,45]. Among them, the species *Habranthus jamesonii* (Baker) Ravenna and *Zephyranthes filifolia* Herb. ex Baker and Kraenzl., collected in San Juan and Mendoza, respectively, were described as the most active against AChE, with IC_50_ values of 1 ± 0.01 and 1 ± 0.08 μg·mL^−1^, respectively, and found to contain galanthamine-type alkaloids among their chemical profiling [45]. Recently, the species of the genus *Eucharis* Planch reported herein have been revised and re-cataloged as *Urceolina* Rchb., as well as *Habranthus* Herb. and *Rhodophiala* C. Presl, which have both been re-named as *Zephyranthes* Herb. [46]. A visual representation of this information is provided in Figure 4, which lists all the reported genera according to the place of collection and using the updated generic names.

### 2.3. Molecular Docking

As shown in Table 2, eight galanthamine-type alkaloids identified in *R. multiflora* were evaluated by molecular docking. Galanthamine, sanguinine, narwedine, 3-*O*-acetylgalanthamine, and 3-*O*-acetylsanguinine have a double bond between C-1 and C-2. Sanguinine, *O*-demethyllycoramine, and 3-*O*-acetylsanguinine show a hydroxyl group at C-9, whereas the other galanthamine-type structures found in *R. multiflora* present a methoxy group at this position. Most of the structures detected in this species have a hydroxyl group at C-3, although alkaloids with carbonyl and acetoxy groups at this position were also found in this plant extract (Figure 2). All the computational assays were carried out on five different x-ray crystals of human acetylcholinesterase (hAChE): 4EY5, 4EY6, 4EY7 [47], 4M0E, and 4M0F [48]. In a molecular docking experiment, the ligands, ions, and water molecules are eliminated from the Protein Data Bank (PDB) file, so the topological form of the active site should be distinguished by the amino acid orientation around the co-crystalized ligand.

Five x-ray PDB structures co-crystalized with different ligands were selected and deleted. Our reference protein was 4EY6, a PDB structure crystalized with galanthamine as the ligand. In this protein, the geometric distribution of a monoacid around the pocket (active site) is optimum for hosting molecules similar to galanthamine. Accordingly, the molecules 3-*O*-acetylgalanthamine, 3-*O*-acetylsanguinine, narwedine, and lycoraminone have higher binding free energy (BE) values than galanthamine (upper 8.75 kcal·mol^−1^).

On the other hand, as molecular docking treats the protein as a rigid body, each hAChE crystal has an active site with a slightly different geometry, relative to each ligand co-crystalized in the x-ray diffraction experiment. For example, in 4EY5, the co-cristallized ligand was huperzine A. The molecular docking results show the same behavior for 4EY6: the alkaloids 3-*O*-acetylgalanthamine, 3-*O*-acetylsanguinine, narwedine, and lycoraminone had the highest BE values. As with 4EY5 and 4EY6, the molecular docking experiments for 4EY7 revealed that 3-*O*-acetylgalanthamine, 3-*O*-acetylsanguinine, narwedine, and lycoraminone had the highest BE values (upper 9.83 kcal·mol^−1^). For 4EY7, the co-crystallized ligand was donepezil.

In the case of 4M0E, the original co-cristallized ligand was dihydrotanshinone I, a molecule with a more planar conformation than galanthamine. The molecular docking experiment showed that lycoraminone will form the most stable ligand–protein complex. For 4M0F, the alkaloid with the lowest BE was 3-*O*-acetylsanguinine; however, the values for the 3-*O*-acetylgalanthamine, narwedine, and lycoraminone molecules were also significantly high (upper 8.74 kcal·mol^−1^). The territrem, a large molecule in a 3D conformation, was the co-crystallized ligand on 4M0F.

**Figure 5:**
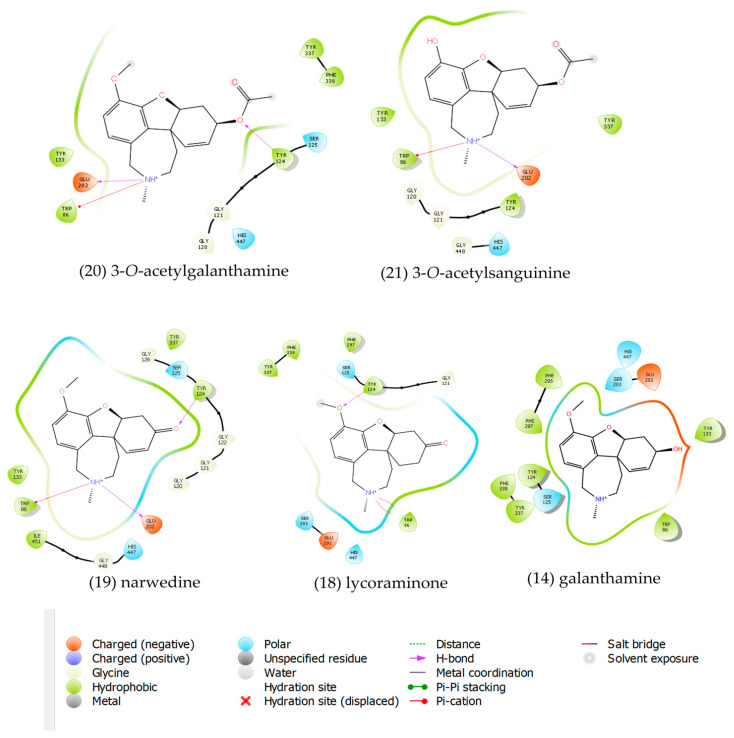
2D ligand–4EY6 protein interaction diagram for 3-*O*-acetylgalanthamine (**20**), 3-*O*-acetylsanguinine (**21**), narwedine (**19**), lycoraminone (**18**), and galanthamine (**14**).

To understand the arrangement of amino acids around the active site in the reference protein 4EY6, a 2D ligand–protein interaction diagram for the most energetically stable alkaloids within the active site was generated and is presented in Figure 5. The stabilization of 3-*O*-acetylgalanthamine (**20**) is produced by the presence of two hydrogen bond interactions with the residues Glu 202 and Tyr 124, one π-cation interaction with Trp 86, and three hydrophobic interactions with Tyr 133, Tyr 337, and Phe 338. For 3-*O*-acetylsanguinine (**21**), the stabilization arises from the presence of one hydrogen bond interaction with Glu 202, one π-cation interaction with Trp 86, and two hydrophobic interactions with Tyr 133 and Tyr 337. For narwedine (**19**), the interactions with the active site are similar to those of compound **20**, with two hydrogen bond interactions with Glu 202 and Tyr 124, one π-cation interaction with Trp 86, and three hydrophobic interactions with Tyr 133, Tyr 337, and Ile 451. Finally, in lycoraminone (**18**), there is one hydrogen bond interaction with Tyr 124, two π-cation interactions with Trp 86, and three hydropohic interactions with Tyr 337, Phe 338, and Phe 297. In conclusion, in all the molecules depicted in Figure 5, the stabilizaton is achieved by the interactions of the NH^+^ group with the residues Trp 86, Glu 202, Ser 203, and His 447 (the last three amino acids known as the catalytic triad) [49]. In the case of galanthamine (**14**), as depicted in Figure 5, the best conformation estimated by the molecular docking experiment shows the NH^+^ group oriented in the opposite direction to the catalytic triad, and this explains the low estimated BE values.

## 3. Materials and Methods

### 3.1. Plant Material Voucher

All the *Rauhia* Traub species, which were collected in Peru over several years, were received as bulbs from botanical gardens and identified by Dr. Alan W. Meerow. The following species have been deposited at the Fairchild Tropical Botanic Garden (FTG) and National Arboretum (NA), both in the USA: *R. multiflora* (Meerow 2441, FTG), *R. decora* (Meerow 1160, FTG), and *R. staminosa* (Meerow 3530, NA).

### 3.2. Extraction

The bulbs of each *Rauhia* species were dried at 40 °C, and then milled. The extraction procedure was carried out according to [50], using fifty mg of each sample to obtain the alkaloid extracts.

### 3.3. GC-MS Analysis

The dried alkaloid extracts of the *Rauhia* species were dissolved in 100 μL of chloroform and analyzed by GC-MS. A total of 1 μL of each sample was injected in a GC-MS 6890N apparatus (Agilent Technologies, Santa Clara, CA, USA) coupled to an Agilent MSD5975 Inert XL, operating in electron ionization (EI) mode at 70 eV, and with a Sapiens-X5 MS column (30 m × 0.25 mm i.d., film thickness 0.25 μm). More information about the chromatographic conditions is available [50].

### 3.4. Alkaloid Identification and Quantification

The chromatograms of each *Rauhia* species were analyzed using AMDIS 2.64 software. The alkaloid profile of each sample was obtained using the library database of the Natural Products Group of Barcelona University (Spain), the NIST 05 Database (Gaithersburg, MD, USA), and by a comparison with the data in the literature. All the alkaloids were quantified through a calibration curve of galanthamine, using codeine as the internal standard.

### 3.5. Enzymatic Assay

The AChE inhibitory activity of each *Rauhia* species was analyzed as described by [50]. The enzyme AChE from *Electrophorus electricus* (Merck, Darmstadt, Germany) was used. The calibration curves of the bulb alkaloid extracts (0.05, 0.1, 0.25, 0.5, 1, and 10 μg·mL^−1^) were applied to obtain the IC_50_ values for the AChE inhibition, using Prism 9 software. Galanthamine was used as a positive control.

### 3.6. Statistical Analysis

The results were analyzed by an ANOVA, using Prism 9 software (Figure 3). The data are expressed as the mean ± standard deviation (SD). Significant results are marked as follows: ** *p* < 0.01, * *p* < 0.1, and *ns* (not significant). One-way ANOVA with Dunnett’s multiple comparison test was used to compare the mean of each column with the mean of a control column (galanthamine).

### 3.7. Molecular Docking

The molecular docking simulations of galanthamine-type alkaloids observed in the species *R. multiflora* were carried out using the Autodock 4.2 program [51]. These in silico experiments require the ligand and protein structures to be correctly prepared. The tridimensional alkaloid structures were downloaded from the PubChem database and were edited using the Maestro program [52] belonging to the Schrodinger suite. In this process, hydrogen atoms were added, and the protonation states were checked for a pH of 7.0 ± 2.0. As a result, nitrogen is protonated in all the evaluated configurations.

Additionally, a set of human AChE protein structures were downloaded from the Protein Data Bank web site and were prepared using the Maestro program. The preparation consisted of deleting the water molecules, ions, and ligands included in the crystallography PDB file. Additionally, the bond orders were assigned, the hydrogen atoms were added, the missing side chains were included, and the amino acid protonation states were checked. In the molecular docking simulations, the first step corresponded to computing a set of pre-calculated grids of affinity potentials via AutoGrid to find suitable binding positions for a ligand on a given macromolecule. In this step, a grid box with dimensions of 60 × 60 × 60 Å and centered in the coordinates −10.30, −43.46, and 30.08 was selected. The second stage in the docking experiment involved obtaining the best orientation of a ligand at the active site of a protein, treated or selected as a rigid body, through the Lamarckian genetic algorithm (LGA) [53]. For this protocol, a population size of 5000 individuals and 50 LGA runs was selected. The best ligand–protein complexes were analyzed according to the potential intermolecular interactions such as hydrogen bonding and the cation–π, π–π stacking.

## 4. Conclusions

This is the first report about the alkaloid profile and biological potential of the genus *Rauhia* Traub (Amaryllidaceae). The most interesting results were obtained for the species *R. multiflora*, which was found to contain several galanthamine-type structures, and its extracts exerted a high in vitro inhibitory activity against AChE. The theoretical interaction of the alkaloids with five different crystallographic structures of human AChE was detailed by in silico experiments. The results indicate that *R. multiflora* is a promising candidate for biotechnological assays to obtain new insights into the biosynthesis of galanthamine-type alkaloids, which may contribute to the development of new methodologies for the sustainable production of galanthamine.

## Figures and Tables

**Table 1:** Alkaloid profile of *Rauhia* species by GC-MS.

Alkaloid	[M]^+^	*m/z*	RI	A ^1^	A ^2^	B ^1^	B ^2^	C ^1^	C ^2^
**Lycorine-type**				**317.5**		**314.5**			
1-*O*-acetylpluviine (**1**)	329 (80)	268 (85), 242 (100)	2598.0	-	-	10.1	0.1	-	-
pluviine (**2**)	287 (78)	286 (52), 268 (55), 243 (61), 242 (100)	2608.2	16.1	1.4	69.2	11.9	-	-
assoanine (**3**)	267 (57)	266 (100), 250 (28), 222 (12), 180 (13)	2622.3	21.6	3.3	24.0	2.9	-	-
norpluviine (**4**)	273 (80)	254 (60), 228 (100)	2635.7	-	-	10.3	0.3	-	-
1-*O*-acetylnorpluviine (**5**)	315 (80)	254 (90), 228 (100)	2641.5	-	-	16.0	2.4	-	-
kirkine (**6**)	273 (<1)	253 (55), 252 (100), 237 (21), 209 (22)	2642.2	13.9	1.4	-	-	-	-
1-*O*-acetyl-9-*O*-methylpseudolycorine (**7**)	345 (30)	284 (25), 242 (100)	2769.3	-	-	10.5	0.3	-	-
galanthine (**8**)	317 (20)	298 (10), 268 (15), 242 (100), 228 (5)	2775.9	21.6	3.3	-	-	-	-
lycorine (**9**)	287 (30)	268 (27), 250 (15), 226 (100), 147 (15)	2789.3	-	-	10.1	0.1	-	-
9-*O*-methylpseudolycorine (**10**)	303 (33)	302 (22), 284 (14), 243 (78), 242 (100)	2830.1	11.4	1.1	17.9	2.4	-	-
sternbergine (**11**)	331 (41)	270 (32), 252 (14), 229 (72), 228 (100)	2844.1	25.8	17.4	10.8	0.6	-	-
2-*O*-acetyl-9-*O*-methylpseudolycorine (**12**)	345 (30)	284 (100), 268 (40), 242 (40)	2907.3	168.9	32.5	135.6	16.6	-	-
2-*O*-acetylpseudolycorine (**13**)	331 (30)	270 (100), 254 (75), 228 (80)	2945.1	38.2	15.4	-	-	-	-
**Galanthamine-type**				**10.0**		**10.8**		**278.7**	
galanthamine (**14**)	287 (94)	286 (100), 270 (25), 244 (42), 216 (49)	2437.0	-	-	-	-	103.6	48.3
lycoramine (**15**)	289 (78)	288 (100), 232 (14), 202 (22), 187 (18)	2459.4	10.0	0.1	10.8	0.4	73.1	10.3
sanguinine (**16**)	273 (100)	272 (81), 256 (23), 230 (16), 202 (44)	2476.2	-	-	-	-	21.7	5.3
*O*-demethyllycoramine (**17**)	275 (67)	274 (100), 218 (8), 174 (13), 173 (17)	2487.6	-	-	-	-	23.4	5.9
lycoraminone (**18**)	287 (68)	286 (100), 244 (5), 218 (17), 202 (23)	2491.6	-	-	-	-	10.3	0.3
narwedine (**19**)	285 (86)	284 (100), 216 (25), 199 (24), 174 (43)	2517.5	-	-	-	-	17.3	1.8
3-*O*-acetylgalanthamine (**20**)	329 (34)	328 (31), 270 (100), 216 (31), 165 (17)	2577.2	-	-	-	-	13.0	0.9
3-*O*-acetylsanguinine (**21**)	315 (46)	256 (100), 255 (59), 254 (40), 212 (29)	2584.6	-	-	-	-	16.3	1.4
**Homolycorine-type**				**57.0**		**42.0**			
nerinine (**22**)	347 (<1)	110 (8), 109 (100), 108 (18)	2511.4	11.0	0.2	18.8	1.6	-	-
homolycorine (**23**)	315 (<1)	110 (11), 109 (100), 108 (30)	2785.4	19.2	2.5	10.0	0.1	-	-
8-*O*-demethylhomolycorine (**24**)	301 (<1)	110 (23), 109 (100), 108 (53)	2847.6	26.8	4.1	13.2	1.3	-	-
**Haemanthamine -type**						**11.0**			
8-*O*-demethylmaritidine (**25**)	273 (100)	230 (24), 202 (27), 201 (93), 189 (60)	2549.8	-	-	11.0	0.4	-	-
**Pretazettine-type**						**11.7**		**9.9**	
*O*-methyltazettine (**26**)	345 (30)	330 (30), 314 (25), 261 (100), 239 (25)	2643.2	-	-	-	-	9.9	0.2
tazettine (**27**)	331 (24)	316 (13), 298 (20), 247 (100), 70 (26)	2686.1	-	-	11.7	1.2	-	-
**Unidentified**				**24.8**		**11.2**			
UI 1 (HLY type) (**28**)	329 (<1)	221 (<1), 109 (100)	2510.8	-	-	11.2	1.1	-	-
UI 2 (HLY type) (**29**)	330 (<1)	221 (<1), 109 (100)	2555.9	13.4	1.2	-	-	-	-
UI 3 (**30**)	325 (40)	282 (100), 266 (10), 139 (60)	2989.5	11.4	0.3	-	-	-	-
**Total:**				**409.3**		**401.2**		**288.6**	

RI: Kovats retention index; UI: unidentified; A: *R. staminosa*; B: *R. decora*; C: *R. multiflora*; HLY type: homolycorine-type; ^1^ values expressed in μg GAL·100 mg^−1^ DW; ^2^ values expressed in %TIC (total ion current).

**Table 2:** Estimated binding free energy in molecular docking studies of alkaloids identified in the species *Rauhia multiflora* toward five different hAChE structures. Values are expressed in kcal·mol^−1^.

alkaloid	4EY5	4EY6	4EY7	4M0E	4M0F
3-*O*-acetylgalanthamine (**20**)	−9.08	−9.77	−11.25	−8.57	−9.93
3-*O*-acetylsanguinine (**21**)	−8.75	−9.76	−10.55	−8.42	−10.11
narwedine (**19**)	−9.15	−9.70	−10.41	−8.69	−9.72
lycoraminone (**18**)	−9.70	−9.48	−9.37	−9.10	−9.25
lycoramine (**15**)	−8.84	−9.08	−8.87	−8.64	−8.41
*O*-demethyllycoramine (**17**)	−8.74	−9.08	−8.91	−8.66	−8.40
sanguinine (**16**)	−8.13	−8.54	−9.14	−8.50	−9.12
galanthamine (**14**)	−8.59	−8.75	−9.83	−7.90	−8.74

## Data Availability

Not applicable.

## Supplementary Materials

The following supporting information can be downloaded at: https://www.mdpi.com/article/10.3390/plants11243549/s1.

## References

[1] World Health Organization–Biodiversity and Health. https://www.who.int/news-room/fact-sheets/detail/biodiversity-and-health.

[2] Newman D., Cragg G.M. (2020). Natural products as sources of new drugs over the nearly four decades from 01/1981 to 09/2019. J. Nat. Prod..

[3] Feher M., Schimidt J.M. (2003). Property distributions: Differences between drugs, natural products, and molecules from combinatorial chemistry. J. Chem. Inf. Comput. Sci..

[4] Lu J.-J., Bao J.-L., Chen X.-P., Huang M., Wang Y.-T. (2012). Alkaloids isolated from natural herbs as the anticancer agents. Evid. Based. Compl. Alt..

[5] Bastida J., Lavilla R., Viladomat F., Cordell G.A. (2006). Chemical and biological aspects of *Narcissus alkaloids*. The Alkaloids: Chemistry and Physiology.

[6] Meerow A.W., Snijman D.A., Kubitzki K. (1998). Amaryllidaceae. Families and Genera of Vascular Plants.

[7] Konrath E.L., Passos C.D.S., Klein-Júnior L.C., Henriques A.T. (2013). Alkaloids as a source of potential anticholinesterase inhibitors for the treatment of Alzheimer’s disease. J. Pharm. Pharmacol..

[8] Traub H.P. (1957). Genus *Rauhia* and *R. peruviana*, gen. & sp. nov. Plant Life.

[9] Ravenna P. (1969). Contribution to South American Amaryllidaceae II. Plant Life.

[10] Traub H.P. (1966). Amaryllid notes, 1966. Plant Life.

[11] Ravenna P. (1978). Contributions to South American Amaryllidaceae VII. Plant Life.

[12] Ravenna P. (1981). Contribution to South American Amaryllidaceae VII [VIII]. Plant Life.

[13] Ravenna P. (2002). New *Rauhia* species from northern Peru. Onira.

[14] Meerow A.W., Nakamura K. (2019). Two new species of Peruvian Amaryllidaceae, an expanded concept of the genus *Paramongaia*, and taxonomic notes in *Stenomesson*. Phytotaxa.

[15] Meerow A.W., Gardner E.M., Nakamura K. (2020). Phylogenomics of the Andean tetraploid clade of the American Amaryllidaceae (subfamily Amaryllidoideae): Unlocking a polyploid generic radiation abetted by continental geodynamics. Front. Plant Sci..

[16] Meerow A.W., Guy C.L., Li Q.B., Yang S.L. (2000). Phylogeny of the American Amaryllidaceae based on nrDNA ITS sequences. Syst. Bot..

[17] Berkov S., Osorio E., Viladomat F., Bastida J., Knölker H.-J. (2020). Chemodiversity, chemotaxonomy and chemoecology of Amaryllidaceae alkaloids. The Alkaloids: Chemistry and Biology.

[18] Heinrich M., Teoh H.L. (2004). Galanthamine from snowdrop—The development of a modern drug against Alzheimer’s disease from local Caucasian knowledge. J. Ethnopharmacol..

[19] Maelicke A., Samochocki M., Jostock R., Fehrenbacher A., Ludwig J., Albuquerque E.X., Zerlin M. (2001). Allosteric sensitization of nicotinic receptors by galanthamine, a new treatment strategy for Alzheimer’s disease. Biol. Psychiatry.

[20] Berkov S., Georgieva L., Boriana S., Bastida J. (2022). Evaluation of *Hippeastrum papilio* (Ravenna) Van Scheepen potencial as a new industrial source of galanthamine. Ind. Crops Prod..

[21] Berkov S., Bastida J., Codina C., de Andrade J.P., Berbee R.L.M. (2013). Extract of Hippeastrum papilio rich in galanthamine. https://patents.google.com/patent/EP2999480B1/en.

[22] Chang X. (2015). *Lycoris*, the basis of the galanthamine industry in China. Res. Rev. J. Agric. Allied Sci..

[23] Nair J.J., Van Staden J. (2014). Cytotoxicity studies of lycorine alkaloids of the Amaryllidaceae. Nat. Prod. Commun..

[24] Nair J.J., Rárová L., Strnad M., Bastida J., Van Staden J. (2015). Mechanistic insights to the cytotoxicity of Amaryllidaceae alkaloids. Nat. Prod. Commun..

[25] Kaur H., Chahal S., Jha P., Lekhak M.M., Shekhawat M.S., Naidoo D., Arencibia A.D., Ochatt S.J., Kumar V. (2022). Harnessing plant biotechnology-based strategies for *in vitro* galanthamine (GAL) biosynthesis: A potent drug against Alzheimer’s disease. Plant Cell. Tiss. Org..

[26] Ortiz J.E., Garro A., Pigni N.B., Agüero M.B., Roitman G., Slanis A., Enriz R.D., Feresin G.E., Bastida J., Tapia A. (2018). Cholinesterase-inhibitory effect and *in silico* analysis of alkaloids from bulbs of *Hieronymiella* species. Phytomedicine.

[27] Šafratová M., Hošt’álková A., Hulcová D., Breiterová K., Hrabcová V., Machado M., Fontinha D., Prudêncio M., Kuneš J., Chlebek J. (2018). Alkaloids from *Narcissus poeticus* cv. Pink Parasol of various structural types and their biological activity. Arch. Pharm. Res..

[28] Hulcová D., Maříková J., Korábečný J., Hošťálková A., Jun D., Kuneš J., Chlebek J., Opletal L., De Simone A., Nováková L. (2019). Amaryllidaceae alkaloids from *Narcissus pseudonarcissus* L. cv. Dutch Master as potential drugs in treatment of Alzheimer’s disease. Phytochemistry.

[29] Cortes N., Alvarez R., Osorio E.H., Alzate F., Berkov S., Osorio E. (2015). Alkaloid metabolite profiles by GC/MS and acetylcholinesterase inhibitory activities with binding-mode predictions of five Amaryllidaceae plants. J. Pharmaceut. Biomed..

[30] Cortes N., Posada-Duque R.A., Alvarez R., Alzate F., Berkov S., Cardona-Gómez G.P., Osorio E. (2015). Neuroprotective activity and acetylcholinesterase inhibition of five Amaryllidaceae species: A comparative study. Life Sci..

[31] Cortes N., Sierra K., Alzate F., Osorio E.H., Osorio E. (2018). Alkaloids of Amaryllidaceae as inhibitors of cholinesterases (AChEs and BChEs): An integrated bioguided study. Phytochem. Anal..

[32] Trujillo-Chacón L.M., Alarcón-Enos J.E., Céspedes-Acuña C.L., Bustamante L., Baeza M., López M.G., Fernández-Mendívil C., Cabezas F., Pastene—Navarrete E.R. (2019). Neuroprotective activity of isoquinoline alkaloids from Chilean Amaryllidaceae plants against oxidative stress-induced cytotoxicity on human neuroblastoma SH-SY5Y cells and mouse hippocampal slice culture. Food Chem. Toxicol..

[33] Moreno R., Tallini L.R., Salazar C., Osorio E.H., Montero E., Bastida J., Oleas N.H., León K.A. (2020). Chemical profiling and cholinesrerase inhibitory activity of five *Phaedranassa* Herb. (Amaryllidaceae) species from Ecuador. Molecules.

[34] Acosta K.L., Inca A., Tallini L.R., Osorio E.H., Robles J., Bastida J., Oleas N.H. (2021). Alkaloids of *Phaedranassa dubia* (Kunth) J.F. Macbr. and *Phaedranassa brevifolia* Meerow (Amaryllidaceae) from Ecuador and its cholinesterase-inhibitory activity. S. Afr. J. Bot..

[35] Tallini L.R., Carrasco A., Acosta K.L., Vinueza D., Bastida J., Oleas N.H. (2021). Alkaloid profiling and cholinesterase inhibitory potential of *Crinum x amabile* Donn. (Amaryllidaceae) collected in Ecuador. Plants.

[36] Soto-Vásquez M.R., Rodríguez-Muñoz C.A., Tallini L.R., Bastida J. (2022). Alkaloid composition and biological activities of the Amaryllidaceae species *Ismene amancaes* (Ker Gawl.) Herb. Plants.

[37] Tallini L.R., Bastida J., Cortes N., Osorio E.H., Theoduloz C., Schmeda-Hirschmann G. (2018). Cholinesterase inhibition activity, alkaloid profiling, and molecular docking of Chilean *Rhodophiala* (Amaryllidaceae). Molecules.

[38] Moraga-Nicolás F., Jara C., Godoy R., Iturriaga-Vásquez P., Venthur H., Quiroz A., Becerra J., Mutis A., Hormazábal E. (2018). *Rhodolirium andicola*: A new renewable source of alkaloids with acetylcholinesterase inhibitory activity, a study from nature to molecular docking. Rev. Bras. Farmacogn..

[39] Fernández-Galleguillos C., Romero-Parra J., Puerta A., Padrón J.M., Simirgiotis M.J. (2022). Alkaloid profiling, anti-enzymatic and antiproliferative activity of the endemic Chilean Amaryllidaceae *Phycella cyrtanthoides*. Metabolites.

[40] Del Rojas-Vera J.C., Buitrago-Díaz A.A., Possamai L.M., Timmers L.F.S.M., Tallini L.R., Bastida J. (2021). Alkaloid profile and cholinesterase inhibition activity of five species of Amaryllidaceae family collected from Mérida state-Venezuela. S. Afri. J. Bot..

[41] De Andrade J.P., Giordani R.B., Torras-Claveria L., Pigni N.B., Berkov S., Font-Bardia M., Calvet T., Konrath E., Bueno K., Sachett L.G. (2016). The Brazilian Amaryllidaceae as a source of aceylcholinesterase inhibitoy alkaloids. Phytochem. Rev..

[42] Gasca C.A., Moreira N.C.S., de Almeida F.C., Gomes J.V.D., Castillo W.O., Fagg C.W., Magalhaes P.O., Fonseca-Bazzo Y.M., Sakamoo-Hojo E., de Medeiros Y.K. (2000). Aceylcholinesterase inhibitory activity, anti-inflammaory, and neuroprotective potential of *Hippeastrum psittacinum* (Ker Gawl.) Herb (Amaryllidaceae). Food Chem. Toxicol..

[43] Ortiz J.E., Pigni N.B., Andujar S.A., Roitman G., Suvire F.D., Enriz R.D., Tapia A., Basida J., Feresin G.E. (2016). Alkaloids from *Hippeastrum argentinum* and their cholinesterase-inhibitory activities: An in vitro and in silico study. J. Nat. Prod..

[44] Zaragoza-Puchol D., Ortiz J.E., Orden A.A., Sanchez M., Palermo J., Tapia A., Bastida J., Feresin G.E. (2021). Alkaloids analysis of *Habranthus cardanasianus* (Amaryllidaceae), anti-cholinesterase activity and biomass production by propagation strategies. Molecules.

[45] Ortiz J.E., Berkov S., Pigni N.B., Theoduloz C., Roitman G., Tapia A., Bastida J., Feresin G.E. (2012). Wild Argentinian Amaryllidaceae, a new renewable source of the acetylcholinesterase inhibitor galanthamine and other alkaloids. Molecules.

[46] García N., Meerow A.W., Arroyo-Leuenberger S., Oliveira R.S., Dutilh J.H., Soltis P.S., Judd W.S. (2019). Generic classification of Amaryllidaceae tribe Hippeastreae. Taxon.

[47] Cheung J., Rudolph M.J., Burshteyn F., Cassidy M.S., Gary E.N., Love J., Franklin M.C., Height J.J. (2012). Structures of human acetylcholinesterase in complex with pharmacologically important ligands. J. Med. Chem..

[48] Cheung J., Gary E.N., Shiomi K., Rosenberry T.L. (2013). Structures of human acetylcholinesterase bound to dihydrotanshinone I and territrem B show peripheral site flexibility. ACS Med. Chem. Lett..

[49] Sierra K., de Andrade J.P., Tallini L.R., Osorio E.H., Yañéz O., Osorio M.I., Oleas N.H., García-Beltrán O., de Borges W.S., Bastida J. (2022). In vitro and in silico analysis of galanthine from *Zephyranthes carinata* as an inhibitor of acetylcholinesterase. Biomed. Pharmacother..

[50] Torras-Claveria L., Berkov S., Codina C., Viladomat F., Bastida J. (2013). Daffodils as potential crops of galanthamine. Assessment of more than 100 ornamental varieties for their alkaloid content and acetylcholinesterase inhibitory activity. Ind. Crops Prod..

[51] Morris G.M., Huey R., Lindstrom W., Sanner M.F., Belew R.K., Goodsell D.S., Olson A.J. (2009). AutoDock4 and AutoDockTools4: Automated docking with selective receptor flexibility. J. Comput. Chem..

[52] (2021). Schrödinger Release 2022-3: Maestro.

[53] Morris G.M., Goodsell D.S., Halliday R.S., Huey R., Hart W.E., Belew R.K., Olson A.J. (1999). Automated docking using a Lamarckinan genetic algorithm and an empirical binding free energy function. J. Comput. Chem..

