# Supplementary material for: Chemical Survey of Three Species of the Genus Rauhia Traub (Amaryllidaceae)
Source: Plants (Basel). 2022 Dec 16;11(24):3549. doi: 10.3390/plants11243549 (PMC9787901; doi:10.3390/plants11243549)
Supplement: Supplementary file 1 [file plants-11-03549-s001.zip › plants-2014666-supplementary.pdf]

Supplementary Material

Chemical survey of three species of the genus *Rauhia* Traub (Amaryllidaceae)

Luciana R. Tallini [1], Edison H. Osorio [2], Strahil Berkov [3], Laura Torras-Claveria [1], María L. Rodríguez-Escobar [1], Francesc Viladomat [1], Alan W. Meerow [4], Jaume Bastida [1,*]

[1] Departament de Biologia, Sanitat i Medi Ambient, Facultat de Farmàcia i Ciències de l’Alimentació, Univer-sitat de Barcelona, Av. Joan XXIII 27–31, 08028 Barcelona, Spain; lucianatallini@gmail.com; lauratorrascl@ub.edu; mrodries116@alumnes.ub.edu; fviladomat@ub.edu; jaumebastida@ub.edu
[2] Facultad de Ciencias Naturales y Matemáticas, Universidad de Ibagué, Carrera 22 Calle 67, Ibagué 730001, Colombia; edison.osorio@gmail.com
[3] Institute of Biodiversity and Ecosystem Research at the Bulgarian Academy of Sciences, Department of Plant and Fungal Diversity, 23 Acad, G. Bonchev Str., Sofia 1113, Bulgaria; berkov@iph.bio.bas.bg
[4] Arizona State University, School of Life Sciences. Tempe, Arizona 85282, USA; ameerow@asu.edu
* Correspondence: jaumebastida@ub.edu

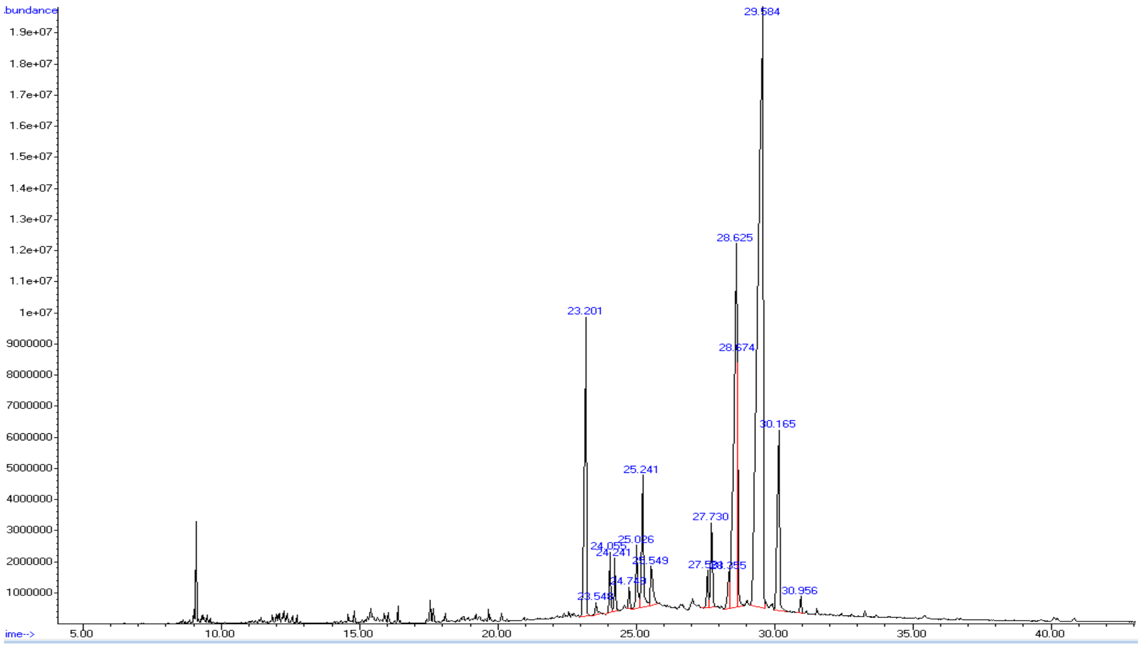

Figure S1. GC chromatogram of the alkaloid extract of *Rauhia staminosa* (sample A).

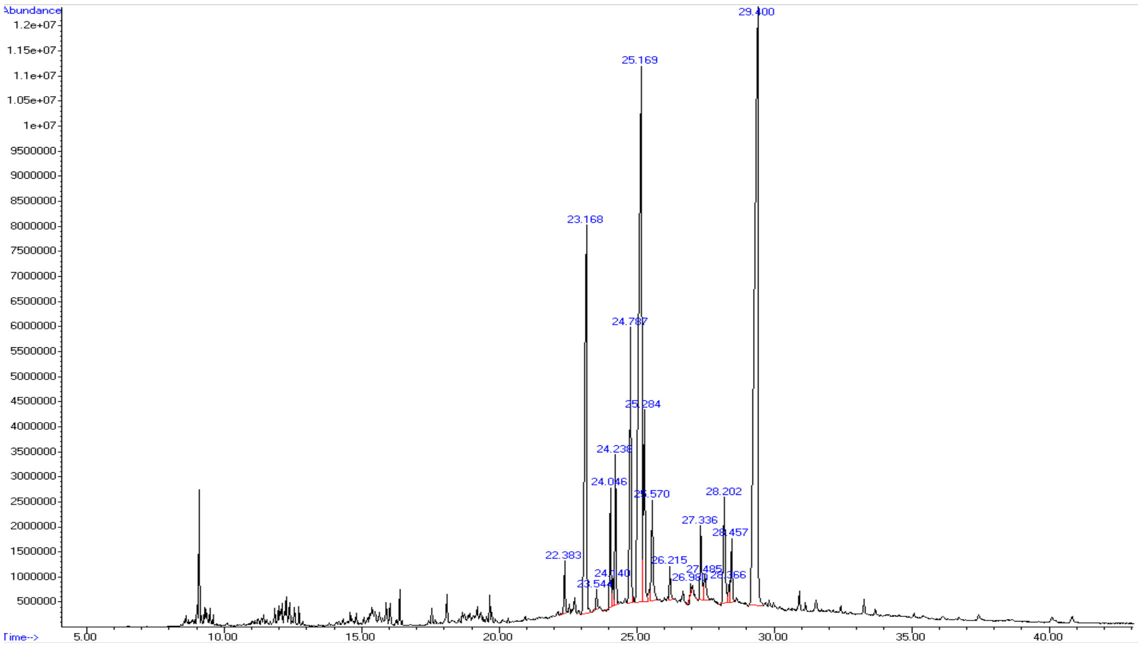

Figure S2. GC chromatogram of the alkaloid extract of *Rauhia decora* (sample B).

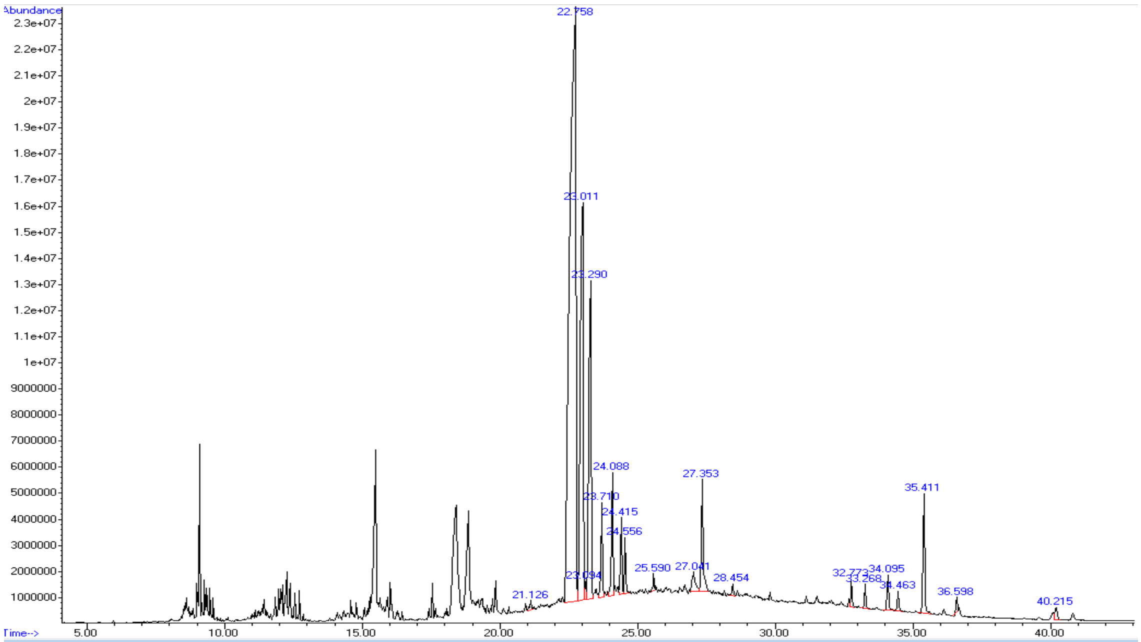

Figure S3. GC chromatogram of the alkaloid extract of *Rauhia multiflora* (sample C)
